# Open Access Increases Citation Rate

**DOI:** 10.1371/journal.pbio.0040176

**Published:** 2006-05-16

**Authors:** Catriona J MacCallum, Hemai Parthasarathy

## Abstract

*PLoS Biology* has published a research article that investigates a bibliometric rather than a biological question: do open-access articles have a citation advantage?

*PLoS Biology* publishes today a research article by Gunther Eysenbach that is not about biology. It is about citations. It provides robust evidence that open-access articles (OA articles) are more immediately recognized and cited than non-OA articles. As such, it adds objective support to the belief we have always held that open-access publication speeds up scientific dialog between researchers and, consequently, should be extended to the whole scientific literature as quickly as possible. It is therefore fitting that we publish such a paper.

We have long argued that papers freely available in a journal will be more often read and cited than those behind a subscription barrier. However, solid evidence to support or refute such a claim has been surprisingly hard to find. Since most open-access journals are new, comparisons of the effects of open access with established subscription-based journals are easily confounded by age and reputation. In the current study, Eysenbach compared citations compiled by Thomson Scientific (formerly Thomson ISI) to individual articles published between June 2004 and December 2004 in the same journal—namely, *Proceedings of the National Academy of Sciences (PNAS)*, which announced its open-access option for authors on June 8 of that year, with an associated publication charge of US$1,000. Non-OA articles in *PNAS* are subject to a six-month “toll-access” delay before the article becomes publicly available. The results of this natural experiment are clear: in the 4 to 16 months following publication, OA articles gained a significant citation advantage over non-OA articles during the same period. They are twice as likely to be cited 4 to 10 months after publication and almost three times as likely between 10 and 16 months. Given that *PNAS* delays open access for only six months, the disparity between OA and non-OA articles in journals where the delay is longer or where articles remain “toll-access” is likely to be even greater.

Eysenbach also looked at the impact of self-archiving non-OA articles. One route to open access, it is argued, is for authors to archive their published articles on their own Web sites or in institutional repositories, although this does not include an explicit business model to cover the cost of peer-review and publishing. The analysis revealed that self-archived articles are also cited less often than OA articles from the same journal.

Yes, you're right; we do have a strong and vested interest in publishing results that so obviously endorse our existence. Moreover, the author of the article is also an editor of an open-access journal. But sometimes a potential conflict of interest can actually help to ensure rigor. In this case, we have an acute interest in ensuring that the article meets the same, if not higher, standards as any other research article we publish. Not only must the conclusions provide a significant advance for the field, but the study must be technically sound, with appropriate evidence to support those conclusions. As with all our research articles, we consulted throughout the evaluation process with an academic editor with appropriate expertise—in this case, Carol Tenopir, professor of information sciences at the University of Tennessee (Knoxville, Tennessee, United States). The article was reviewed by two experts in bibliometric analyses and information science, and an experienced research biologist with expertise in statistics. They all enthusiastically supported publication, although one understandably questioned the suitability of *PLoS Biology* as the publication venue.

We have no intention of making *PLoS Biology* a regular home for bibliometric studies (even when about open access). What makes this study worth publishing in *PLoS Biology* is not only the relative strength of evidence supporting the claim but also the extent to which many (especially other publishers) have anticipated such an analysis. As far as we are aware, no other study has compared OA and non-OA articles from the same journal and controlled for so many potentially confounding factors. Eysenbach's multivariate analysis took into account the number of days since publication, number of authors, article type, country of the corresponding author, funding type, subject area, submission track ( *PNAS* has three different ways that authors can submit a paper), and the previous citation record of the first and last authors. He even administered a supplementary questionnaire to assess whether authors choosing the OA option in *PNAS* chose to do so for only their most important research (they didn't). As Ian Rowlands from the Centre for Publishing at University College London—and one of the reviewers who agreed to be identified in this article—said at the start of his review:

*“Many (most) of the papers and presentations I have read/seen on this topic have completely failed to address the kinds of confounding issues that are so convincingly tackled here. For that reason alone, this paper deserves to be published and alerted to the widest possible audience.”*

In addition to providing evidence for the immediate advantage of open access, Eysenbach's analysis also highlights several potential challenges to its long-term future. Although a limited dataset, the citation history of the first and last authors differed between those who chose the open-access option and those who did not. In the group that chose open access, last authors tended to have a “stronger” previous citation record, whereas this situation was reversed among the group that declined the open-access option—here, it was the first authors who tended to be stronger. This may reflect varying attitudes of authors at different stages of their career, a stronger influence from the leader of a particular group, or an age- or career-related difference in the ability to pay the publication charge (e.g., [ 1]). Indeed, access to appropriate funds may also be a reason why a lower proportion of authors from European countries tended to choose the open-access option. In many of these countries, funds for page charges—and, by extension, open-access publication fees—are often not included within research grants.

*PNAS* was one of the first journals to offer an open-access option to its authors. However, such hybrid journals are increasing: Blackwell, Springer, and Oxford University Press now provide this option as well. This means that similar experiments can be replicated. Moreover, although the evidence from the current analysis argues most strongly for a time advantage in citation for OA articles, a study over longer periods will reveal whether this translates into a sustained increase in the number of citations. In the meantime, open-access advocates should be emboldened by tangible evidence for what has seemed obvious all along.

## Abbreviations

OA article: open-access article
PNAS: *Proceedings of the National Academy of Sciences*
